# Key findings from 15 years of *Mangrovibacter* research: a generalist bacterium beyond endophytes

**DOI:** 10.1128/aem.02479-24

**Published:** 2025-07-10

**Authors:** Hong Soon Chin, Narendrakumar Ravi Varadharajulu, Kah Cheng Teo, Peter Chiew Hing Cheong, Sen-Lin Tang

**Affiliations:** 1 Institute of Bioinformatics and Structural Biology, National Tsing Hua University 597945, Hsinchu, Taiwan; 2 Viroxy Sdn Bhd, Kuala Lumpur, Malaysia; 3 Department of Chemistry, National Tsing Hua University 150417, Hsinchu, Taiwan; 4 Department of Agricultural and Food Science, Universiti Tunku Abdul Rahman 65285, Kampar, Malaysia; 5 Biodiversity Research Center, Academia Sinica 71566, Taipei City, Taiwan; Universidad de los Andes, Bogotá, Colombia

**Keywords:** *Mangrovibacter*, *M. phragmitis*, *M. plantisponsor*, *M. yixingensis*, endophytes, zoonotic, symbiotic, bioremediation, mode of transmission

## Abstract

Since the discovery of *Mangrovibacter plantisponsor* in 2010, research on *Mangrovibacters* (MGBs) has stagnated. Although laboratories worldwide have isolated various MGB strains and deposited their 16S rDNA sequences in the NCBI database, a limited understanding of MGBs has resulted in only a few publications from these collections. Recent advancements in metagenomic technology have revealed the presence of MGBs in a broader range of habitats. Most microbiomes exhibit low MGB abundance (typically <1%). Even in environments with higher prevalence, such as salt-tolerant aerobic granular sludge (75%), the gut of superworms fed with polyurethane (22%), or fermented foods like mandai (16%), the functional roles of MGBs remain unclear. Through meticulous curation of publications and data from MicrobeAtlas and AMIBASE, MGBs can be classified as free living, endophytic, or zoonotic. Recent evidence suggests their presence in food sources and potential interactions with humans. Current studies confirm the coexistence of MGBs with humans. This review underscores the phenotypic features and genomic foundations of MGBs, highlighting attributes such as endophytic behavior, diverse metabolite utilization, tolerance to salinity and pH, metal homeostasis, biofilm formation, and bioremediation potential. Insights are derived from the analysis of four MGB genomes deposited in NCBI since 2014, along with three newly reported genomes in 2024. Experimental and genetic evidence suggests that MGBs act as “generalist microbes” capable of thriving in diverse nutrient sources and harsh environments. This review elucidates prospective research trajectories and highlights numerous potential commercial applications of MGBs, emphasizing the need for further investigation into their roles and benefits.

## INTRODUCTION

Since 2010, only three valid species of *Mangrovibacter* (MGB) have been published. The genus MGB within the *Enterobacteriaceae* family comprises *M. phragmitis* (MPH), *M. yixingensis* (MYI), and *M. plantisponsor* (MPL). The strains MPH MP23^T^, MYI TULL-A^T^, and MPL MSSRF40^T^ were isolated from the roots of *Phragmites karka* (1), farmland soil (2), and mangrove-associated wild rice plants (3), respectively. These strains are facultative anaerobes and Gram negative, exhibiting plant growth-promoting and nitrogen-fixing (NF) properties. MGBs are understood to be ubiquitous in coastal ecosystems worldwide, enhancing microbially mediated biogeochemical processes (4). Increasing evidence suggests that MGBs are more widely distributed across ecosystems than previously recognized (see Table 1). Current data indicate that MGBs can be classified as “generalist microbes” due to their broad environmental tolerances (5). MGBs are known to thrive across a wide pH range of 4 to 10 and can survive in media with up to 20% NaCl (Table 2), classifying them as halotolerant bacteria. Notably, the MYI strain T1 (China patent no. CN113913329B) can survive in wastewater with high NaCl concentrations (≤200 g/L) and a high chemical oxygen demand (COD) of 10,000 mg/L. Other traits that enable MGBs to endure diverse habitats and harsh conditions include flexible carbon source utilization and extensive metal ion homeostasis. This review also underscores the pivotal role of MGB in the domains of bioremediation and biodegradation, as well as in the enhancement of plant growth and food processing.

**TABLE 1:** Distribution of MGB in various environments^*a*^

Group	Source	Species/genus	Technique/ abundance	Ref.	Origin	Remarks
Free living	Salt-tolerant sludge	MPH ASIOC01	V3–V4 and FL metagenomic/<1%/WGS (PacBio)	(6)	Taiwan	Genome available; GenBank assembly no. GCA_040429585.1
	Sequencing batch reactor in anaerobic/aerobic mode (R2)	MGB	V3–V4 metagenomics/3.2%	(7)	China	MGB might be responsible for EPS secretion
	Aerobic granular sludge (AGS)	MGB	V3–V4 metagenomics/ Up to 35.9%	(8)	China	High abundance when added acyl-homoserine lactone (AHL)
	Salt-tolerant AGS	MGB	V3–V4 metagenomics/ Up to 74.9%	(9)	China	High abundance of MGB detected at initial stage of AGS formation
	Olive mill wastewater sludge	MGB	V4 variable region PCR primer metagenomics	(10)	Morocco	Certain genera, like MGB sp., disappeared by the end of treatment
	Non-acclimated sludge (non-AS) and acclimated sludge	MGB	Metagenomics/ up to 45.7%	(11)	China	Acclimated sludge containing 70 g/L of NaCl (AS_70); abundance of MGB in the AS_70 group (45.7%) was significantly higher than in the non-AS_70 group (22.3%)
	Acidogenic fermentation under salt stress	MGB	Metagenomics	(12)	China	MGB decreased significantly with the exogenous addition of AHLs, showing a negative correlation between MGB and AHLs; in contrast, the relative abundance of MGB increased with the addition of quorum-quenching agents (curcumin)
	Upflowed fixed-film microaerophilic–aerobic bioreactor (UFMB) system treating raw textile effluent	MGB	V3–V4 metagenomics/2.06% after 1-day hydraulic retention time in the upper zone of the UFMB	(13)	India	Genus-level distribution of the established biofilm consisted of MGB and other bacteria
	Anaerobic sequencing batch reactor	MGB	MGB-porin OmpA	(14)	Netherlands	Identified proteins incorporated ^13^C-label supplied by the proteinaceous substrate in the label protein incubation batch, indicating that MGB is a potential protein degrader
	Tannery effluent sludge	MPL CR1	16S rRNA sequencing	(15)	China	Cr (VI) reduction
	Tannery effluent (semi-solid soil)	MYI MS 2.4	16S rRNA sequencing	(16)	India	Cr (VI) reduction
	Textile effluent	MYI AKS2	16S rRNA sequencing	(17)	Bangladesh	Bioremediation of BR-18 dye
	Natural gas wastewater treatment unit	Maybe MPH and MPL	16S rRNA sequencing	(18)	Egypt	Potential for bioremediation
	Blackish water	MPL UMTKB3	16S rRNA sequencing	(19)	Malaysia	MPL produced PHA when grown in a medium with glucose as the carbon source, and PHA production was confirmed using gas chromatography
	Soil sediment from brackish water	MGB sp. AB6	16S rRNA sequencing	NA	India	Accession no. JX188076.1
	Mangrove rhizosphere sediment	MYI SaN21-3	Isolation and WGS (Illumina)	NA	China	Genome available; GenBank assembly no. GCA_020523985.1
	Farmland soil	MYI TULL-A^T^	16S rRNA sequencing	(2)	China	MYI type strain
	Copper-containing wastewater	MGB sp. A1	16S rRNA sequencing	NA	Taiwan	Accession no. MT762841.1
	Sewage	MGB sp. WG-1	16S rRNA sequencing	NA	China	Accession no. FJ404760.1
	AS from oil field wastewater treating system	*Salmonella*/MGB sp. ZZ-4	16S rRNA sequencing	NA	China	High ANI similarity to MGB suggests that it may not be a *Salmonella* species; accession no. FJ667502.1
	Shrimp aquaculture farm	MGB sp. MFB070	Isolation and WGS (Illumina)	(20)	India	Genome is available and may belong to MPL; GenBank assembly no. GCA_000705335.1
	Automotive biodiesel	Enterobacteriaceae bacterium JW72.7a	DGGE, 16S rRNA gene V6 amplicon pyrosequencing and genotyping/ 5%	(21)	UK	Contaminant bacteria in the biodiesel tank showed high 16S rDNA sequence similarity to MGB
	Geothermal lake	MYI CEMTC 1393	16S rRNA sequencing	NA	Russia	Available from the Collection of Extremophiles and Type Cultures (CEMTC); accession no. OP143686.1
	Pond (“Shoubuike”)	MGB	Shotgun metagenomic sequencing	(22)	Japan	MGB tended to have a significant impact on other bacteria within the metabolic interaction networks during the time-series experiment; MGB MAG no. MTS-040_bin.003
	Water drop of natural oil-emitting lake	MGB	Single-cell sequencing	(23)	Trinidad and Tobago	Possibly involved in the anaerobic biodegradation of polycyclic aromatic hydrocarbons (HCs)
	HC-containing system	MGB	Next-generation sequencing/0.2%	NA	USA	An HC-containing system comprising a water injection system, an HC extraction system, and an HC production system; relative abundance of MGB after treatment with diphenyliodonium chloride for 305 days was 0.2%; U.S. Patent Application Ser. no. 62/111,797
	Florida oak spring	MGB	Metabarcoding of the 16S rDNA and *rbcL* regions	(24)	USA	High-sulfur, low-oxygen environments created by underwater sinkholes and springs form unique habitats populated by microbial mat communities
	Soil	MGB	Culture collection	NA	Indonesia	Indonesian Culture Collection; code No. InaCC B841 (ASEAN Microbial Database)
	Hydraulic fracturing fluids	MPL	16S rRNA sequencing	(25)	USA	Frack water source: treated and grown at 37°C using ONR7a media supplemented with Angola oil and peptones
Endophytes/associated with plants/food	Roots of *Phragmites karka*	MPH MP23^T^	Isolation and WGS (Illumina)	(1)	India	Type strain and genome available; GenBank assembly no. GCA_001655675.1
	*Spartina alterniflora* (salt-water cordgrass)	MPL	Morphospecies Sanger sequencing	(26)	USA	Root endophyte; MPL average abundance UNOILED REFERENCE: 0.33 MPL average abundance OILED: 0.17
	Mangrove litterfall	MGB sp. SLW1	Isolation and WGS (Oxford Nanopore/ONT)	(4)	India	Genome is available and belongs to MYI; GenBank assembly no. GCA_039652315.1
	Rhizosphere soils of *Avicennia marina*	MPL and MYI	16S rRNA sequencing	(27)	India	2 MPL and 1 MYI isolate
	Rhizosphere soils of *Rhizophora mucronata*	MPL and MYI	16S rRNA sequencing	(27)	India	1 MPL and 1 MYI isolate
	Intersecting regions of *Avicennia marina* and *Rhizophora mucronata*	MPL and MYI	16S rRNA sequencing	(27)	India	1 MPL and 1 MYI isolate
	Rhizosphere soils of *Suaeda maritima*	MPL	16S rRNA sequencing	(27)	India	1 MPL isolate
	Rhizosphere soils of *Salicornia brachiata*	MPL	16S rRNA sequencing	(27)	India	2 MPL isolates
	*Cerbera manghas* L. (Sea mango)	MPL	16S rRNA sequencing	NA	China	Isolate from the leaf, stem, and root of *Cerbera manghas* L; diversity of endophytic and rhizospheric bacteria isolated from semi-mangrove *Cerbera manghas* L. (28)
	*Porteresia coarctata Tateoka* (mangrove-associated wild rice)	MPL MSSRF40^T^	Isolation and WGS (Illumina)	(3)	India	Type strain and genome available; GenBank assembly no. GCA_003182475.1
	Mangrove rhizosphere/root	MPL MSSRF N80 and MPL BCRP5	16S rRNA sequencing	NA	India	Accession no. KU131265.1 and MT422011.1; other isolates obtained: MGB sp. MSSRF N44, MSSRF N87, MSSRF N97, MSSRF N107, P4, P5, P6, P7, P12, P13, P16, P21, and *Klebsiella*/MGB sp. P23
	Todos os Santos Bay oil-contaminated mangrove	MGB sp. QUEBA02 and 03	16S rRNA sequencing	NA	Brazil	Potential HC-degrading bacterium isolated from sediment in a Brazilian mangrove; accession no. JQ658399.1 and JQ658400.1
	*Bruguiera sexagula* (upriver orange mangrove) and *Ceriops decandra* (yellow mangrove)	NVVC2a and BDMO2b	16S rRNA sequencing	(29)	Vietnam	Growth media were supplemented with 2% NaCl and may be associated with MPL
	*Rhizophora stylosa* (bakau pasir)	MGB	Isolation/17.6%	(30)	China	MGB was isolated from the stem, root, leaf, and other tissues of *Rhizophora stylosa*
	Rhizosphere soil	MGB	V3–V4 metagenomics	(31)	USA	Root endophytic microbes
	*Oryza sativa* (rice)	MYI	Culture collection	NA	Thailand	Thailand Bioresource Research Center, code no. TBRC 3517 (ASEAN Microbial Database)
	*Eleocharis dulcis* (water chestnut)	MGB	Culture collection	NA	Thailand	Thailand Bioresource Research Center, code no. TBRC 2986, 2987, and 3518 (ASEAN Microbial Database)
	*Ginkgo biloba* leaves	MGB	V4 region (799F and 1115R) metagenomics/<1%	(32)	China	MGB was detected but showed no association with flavanol glycosides
	*Euterpe oleracea* (açai) fruits	MGB	V4 region (341F and 805R) metagenomics/<1%	(33)	Brazil	MGB was detected during the spontaneous decay process of açaí fruits
	Pulped natural fermented coffees	MGB	V3–V4 metagenomics	(34)	Brazil	Distinctive genera of MGB were detected at an altitude of 800 m
	Pericarp of *Garcinia mangostana* (mangosteen)	MPL 21A1 and 21A3	16S rRNA sequencing	(35)	Malaysia	MGB was detected after 20 to 30 days of fermentation
	Soy sauce mash	MGB sp. MFB070	Soy sauce metagenome	NA	Malaysia	Metagenomic sample from soy sauce mash during fermentation process; BioProject no. PRJNA185981
	Spoiled green manzanilla Spanish-style table olive fermentations	MGB	V3–V4 metagenomics	(36)	Spain	Detected in samples from spoiled green manzanilla Spanish-style table olive fermentations in Industry A
	1-Methylcyclopropene (1MCP)-treated and directly brined olives (DB)	MGB	V3–V4 metagenomics DB-Control: 2.78% DB-1MCP: 1.88%	(37)	Spain	Olive samples from each fermentation vessel were collected at the end of fermentation (176 days), washed in sterile water, and pitted under sterile conditions before DNA extraction
	*Cajanus cajan* L. Millsp (pigeon pea)	MGB	Oxford Nanopore Technologies/ full-length 16S rDNA	(38)	Indonesia	Metataxonomic analysis of bacterial diversity in pigeon pea after soaking in water
	Pre-fermented coconut water	MGB	V3–V4 metagenomics	(39)	China	MGB and a few other genera of bacteria emerged as biomarkers that distinguished the pre-fermented coconut water prepared in March from those prepared in April, May, June, and July
	Kimchi (fermented napa cabbage)	MPL	16S rRNA amplicon sequencing Set 1 (V2, V4, and V8) Set 2 (V3, V6 + V7, and V9)	(40)	Korea	ISO method MPL 15.93%, SMA method MPL 10.43%; ISO and SMA methods resulted in more selective enrichment of Enterobacteriaceae with the addition of 20 mg/L of novobiocin
	Mandai (a fermented food prepared using the inner flesh of jackfruit)	MGB	Metagenomics/15.5%	(41)	Indonesia	Products were spontaneously fermented using only salt and stored in sealed containers
Zoonotic/associated with human	Gut of *Zophobas atratus* (giant mealworm beetle) larvae	MGB	V3–V4 metagenomics/up to 21.88%	(42)	China	Polyurethane (PU)-fed superworms; larvae of *Zophobas atratus* are commonly known as superworms
	Superworms (*Zophobas atratus*)	MGB	V3–V4 metagenomics	(43)	Korea	Gut microbiota of larvae fed with polyvinyl chloride (PVC) included various new groups of bacterial genera, including MGB
	*Rhynchophorus phoenicis* (African palm weevil)	MGB	V3–V4 metagenomics/0.1%	(44)	Nigeria	MGB was detected in the gut microbiota of African palm weevil larvae
	Frass of *Hermetia illucens* (black soldier fly), *Tenebrio molitor* (yellow mealworm), and *Gryllus assimilis* (Jamaican field cricket)	MGB	V4 region metagenomics	(45)	Australia	Frass is defined as insect excrement, with microorganisms primarily introduced through insect feces; frass is commonly used as an organic fertilizer
	Dog (brachycephalic breeds)	MGB	V1–V3 metagenomics	(46)	Belgium	Brachycephalic breeds have a distinct nasal microbiota profile, with higher proportions of MGB compared to meso- and dolichocephalic dogs
	Great tit (*Parus major*)	MGB	Metagenomics	(47)	Czech Republic	Great tit individuals on a mixed diet experienced significant increases in the relative abundance of MGB; mixed diet consists of seeds, mealworms, and fruits
	Broiler (chicken)	MGB	V3–V4 metagenomics	NA	China	MGB was enriched in the 21-day-age control group (*P* < 0.05); effects of phytosterols on growth performance, ileal morphology and microbial flora structure of broiler (48)
	Cecum of chicken (*Gallus gallus domesticus*)	MGB	Set 1 (V2, V4, and V8) and Set 2 (V3, V6 + V7, and V9)/0.01%–0.4%	(49)	South Africa	Chicken cecal microbiota
	Diarrheic animals (calves and buffalo) ranging in age from newborn to 3 months	MGB sp. NCCP-463	16S rRNA sequencing	(50)	Pakistan	Presence of multidrug-resistant bacteria in the fecal microbiota of neonatal calves with diarrhea (NCD)
	Intestine of juvenile largemouth bass (*Micropterus salmoides*)	MGB	V4–V5 metagenomics/5.88%	(51)	China	MGB was detected in the intestines of juvenile largemouth bass when their feeding was supplemented with methionine hydroxy analog
	Brown-marbled grouper (*Epinephelus fuscoguttatus*)	MGB	V3–V4 metagenomics	NA	China	A total of 19,838 specific OTUs were detected in the gut of grouper, with MGB and other bacterial taxa being the primary genera in the population; bacterial diversity analysis of recirculating aquaculture *Epinephelus fuscoguttatus* (52)
	Intestine of shrimp	MGB	V4 region metagenomics/4.99%	(53)	China	*Lactobacillus* (11.08%), MGB (4.99%) and *Fusibacter* (2.18%) were the dominant genera in the intestines of shrimp
	Pine wood nematode (*Bursaphelenchus xylophilus*)	MGB sp. Arv-29-1.1a	1.7% (MGB % frequency from *P. nigra*)	(54)	Portugal	Frequencies of MGB associated with *B. xylophilus* isolated from *Pinus nigra*
	*Euryphorus nordmannii*	MYI JvC08	RAPD typing and sequencing	NA	India	MGB was present in parasitic copepods and isopods; accession no. OL347579.1
	Stomach and hindgut of Tioman crab (*Neosarmatium indicum*)	MGB sp. Nin4-HG	16S rRNA sequencing: stomach 33.3%, hindgut 33.3%	(55)	Hong Kong	Prominent cellulolytic bacteria were isolated from the gut of crabs along the land–sea transition
	Midgut or hindgut of violet vinegar crab (*Episesarma versicolor*)	MGB sp. Eve2-HG	16S rRNA sequencing: midgut 14.8%, hindgut 28.4%	(55)	Hong Kong	Prominent cellulolytic bacteria were isolated from the gut of crabs along the land–sea transition
	Lung microbiota of checkpoint inhibitor pneumonitis (CIP) patients	MGB and MPL	V3–V4 metagenomics	(56)	China	MPL wasmore abundant in the CIP group than in the idiopathic pulmonary fibrosis (IPF) group; it remains unclear whether MGB is pathogenic
	Human sputum	MPH strain PSU-3885-11	Isolation and WGS (MGI Tech)	(57)	Thailand	Submitted by Faculty of Medicine, Prince of Songkhla University, Thailand; GenBank assembly no. GCF_041227045.1; it remains unclear whether MGB is pathogenic
	Obese patients and normal weight subjects with functional dyspepsia (gastric bacteria)	MGB	V3–V4 metagenomics	(58)	Cyprus	MGB, with an abundance greater than 1%, may be considered a typical gastric bacterium; it remains unclear whether MGB is pathogenic
	Human fecal samples	MGB	V1–V2 metagenomics	(59)	Germany	Detected in Grey module PopGen data set and Grey module FoCus data set; it remains unclear whether MGB is pathogenic
	Human midstream urine (healthy cohorts and urinary tract infections cohorts)	MGB	Shotgun meta-transcriptomics	(60)	Germany	It remains unclear whether MGB is pathogenic
	Human tear film (Sjogren’s and non-Sjogren’s aqueous deficiency dry eye)	MGB	V3–V4 metagenomics	(61)	India	It remains unclear whether MGB is pathogenic
	Human fecal samples (health control and patient associated with severe alcoholism)	MPL	V2–V9 metagenomics	(62)	India	It remains unclear whether MGB is pathogenic

^*a*^ NA indicates not available.

**TABLE 2:** Details of the genome assembly and its basic features

	*M. phragmitis*	*M. phragmitis*	*M. phragmitis*	*M. plantisponsor*	*Mangrovibacter* sp.	*M. yixingensis*	*M. yixingensis*	*Mangrovibacter* sp.
GenBank assembly	GCA_040429585.1	GCA_001655675.1	GCA_041227045.1	GCA_003182475.1	GCA_000705335.1	GCA_020523985.1	NA^*c*^	GCA_039652315.1
Stain ID	ASIOC01	MP23^T^	PSU-3885-11	MSSRF40^T^	MFB070	SaN21-3	TULL-A^T^	SLW1
NCBI taxon ID	1691903	1691903	1691903	451513	1224318	1529639	1529639	3144848
Origin of strain	Activated sludge	Roots of *Phragmites karka*	Sputum of *Homo sapiens*	Roots of *Porteresia coarctata* Tateoka	Shrimp aquaculture farm/pond	Mangrove rhizosphere sediment	Farmland soil	Mangrove *Avicennia alba* litterfall
Living habitat	Free living	Endophyte	Zoonotic	Possibly endophyte	Free living	Free living	Free living	Free living
Growth pH	7.5	5–10 7.0 (optimal)	NA	7.0	NA	7.6	4.0–9.0, optimally at 7.0–8.0	4.74–9.32
Salt tolerance (NaCl % wt/vol)	0%–11%, 3% (optimal)	0%–8%, 1% (optimal)	NA	0%–8%, no growth at 10%	NA	~2.0%	0%–6%; 0% (optimal), no growth at 7%	Salinity of 20 6.18% (wt/vol predicted)
Assembly level	Complete	Contig	Contig	Scaffold	Scaffold	Contig	NA	Contig
Contigs^*a*^	1	50	52	56	57	25	NA	27
GC content^*a*^	50.39	49.91	50.03	50.43	50.4	49.66	52 mol%	49.5
Contig L50^*a*^	1	4	7	7	8	2	NA	1
Genome length^*a*^	5,765,145 bp	4,947,475 bp	5,035,050 bp	5,352,990 bp	5,361,575 bp	4,983,067 bp	NA	5,533,946 bp
Contig N50^*a*^	5,765,145	428,946	207,029	356,458	222,077	684,126	NA	3,259,965
Hypothetical proteins^*a*^	1,855	1,308	1,444	1,422	1,488	1,170	NA	2,467
Proteins with functional assignments^*a*^	4,107	3,673	3,718	3,994	3,947	3,790	NA	5,788
Total number of pathways predicted^*b*^	396	263	386	390	376	381	NA	232
Sequencing technology	PacBio Sequel	Illumina MiSeq	MGISEQ2000	Illumina HiSeq	Illumina MiSeq	Illumina	NA	MinION Oxford Nanopore
Assembly method	Flye v. 2.7	MaSuRCA v. 3.1.3	Shovill v. 1.1.0	SPAdes v. 3.10.1	ABySS v. 1.3.7	SPAdes v. 3.13.79	NA	Flye v. 2.9.3
Deposited date	July 2024	June 2016	August 2024	May 2018	June 2014	October 2021	NA	May 2024
Type strain	MP23^T^	MP23^T^	MP23^T^ (based on *trpB*)	MSSRF40^T^	MFB070/MSSRF40^T^	TULL-A^T^	TULL-A^T^	TULL-A^T^ (based on *trpB*)

^*a*^ Predicted with PATRIC v3.6.12.

^*b*^ Predicted with Pathway Tool v26.0.

^*c*^ NA indicates not available.

## DISTRIBUTION OF MGBs

**Fig 1:**
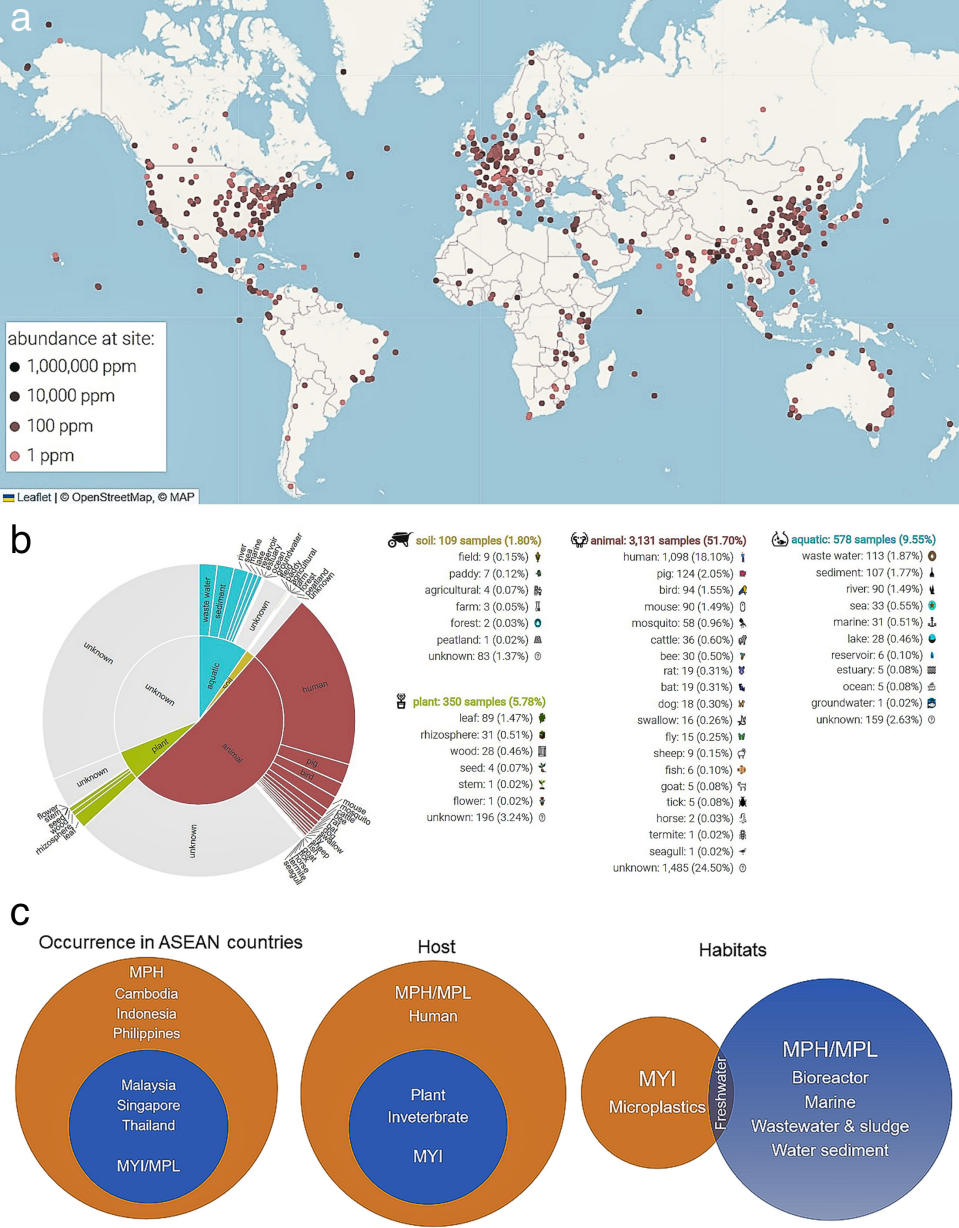
Distribution and habitats of MGBs. (**a**) Observed locations of MGB determined via MicrobeAtlas 1.0, with a similarity level set at 98%; (**b**) habitat and sub-habitat of MGB determined via MicrobeAtlas 1.0, also with a similarity level set at 98%; and (**c**) analysis of occurrence, host, and habitat for MGB using information from the AmiBase database.

We previously noted that MGB cultures were predominantly obtained from East Asia, Southeast Asia, and South Asia (6). However, the availability of newer metagenomic data and the advance of more sophisticated analytical tools, such as MicrobeAtlas 1.0 (Microbe Atlas Project) (63), suggest that this notion may be outdated. Emerging evidence indicates the widespread global distribution of MGBs (Fig. 1a) and their presence in diverse habitats (Fig. 1b). It is notable that the AmiBase database (https://www.amibase.org) houses a comprehensive collection of MGB isolates and metagenomic data (64). By summarizing the information available in AmiBase, we can more effectively consolidate the habitats and hosts of MGBs (Fig. 1c). Both platforms indicate that humans are key hosts for MGBs. Based on the curated data presented in Table 1, we can classify MGBs into free-living, endophytic, and zoonotic categories. This minireview incorporates recent publications alongside earlier reports (6), focusing particularly on the presence of MGBs in food and human contexts. We did not identify any clear habitat preferences among these MGB species. This question can only be accurately addressed once the species identities of MGB isolates are clearly defined, as most have only been reported at the genus-level classification.

## ISOLATION APPROACHES AND SPECIES DELIMITATION METHOD

Currently, there are no established gold standards for isolating MGBs. We have previously consolidated and suggested potential solutions (6, 65) including the use of nutrient-rich media such as Bacto Marine Broth, LB broth supplemented with 15% (vol/vol) glycerol (2), LB agar (1), brain heart infusion agar (16), and a medium consisting of meat extract, yeast extract, and peptone (19). However, these nutrient-rich media are often unsuitable as they fail to effectively eliminate undesirable microbial flora.

A more effective strategy for strain separation relies on the distinctive physiological and biochemical characteristics of MGBs. These NF bacteria can grow independently and thrive in nitrogen-depleted environments, making them suitable for isolation using Burk’s N-free medium supplemented with 2% NaCl (29) or an N-free medium with added yeast extract (NfM + Y agar) (3). Additionally, MGBs can be isolated using NBRIP medium, developed by the National Botanical Research Institute in Rana Pratap Marg, India, and supplemented with 2% NaCl (29, 66). The NBRIP medium was initially formulated to screen for phosphate-solubilizing bacteria (PSB). Given their ability to reduce Cr(VI), MGBs can also be cultivated on LB agar plates that are supplemented with Cr(VI) (15, 16).

Uniquely, MPH ASIOC01 was isolated from acrylonitrile-rich sludges enriched with 1,3-dichloro-2-propanol (1,3-DCP) and 3-chloro-1,2-propanediol (3-MCPD) as carbon sources (6), while MGB sp. SLW1 was isolated using tannic acid agar medium (0.5% wt/vol) with 20% salinity and pH 7.6 (4). Notably, the introduction of quorum quenchers, such as curcumin (12), signaling molecules, such as acyl homoserine lactone (8), or antibiotics, such as novobiocin (40), could enhance the MGB population in the microbiome. By leveraging these unique features of MGBs, we can combine these selection pressures to facilitate their isolation.

Due to challenges in taxonomically distinguishing MGB strains based on similar 16S rDNA sequences, the housekeeping genes *trpB* and *pepN* have been established as valuable markers for efficiently delineating MPH, MYI, and MPL (6). Whole-genome sequencing (WGS) data can further aid in determining the taxonomic classification of MGBs at the species level using overall genome-related index (OGRI) analysis (6, 67). Unfortunately, there is a lack of genus-specific detection methods for MGBs, such as PCR, enzyme-linked immunosorbent assay, biochemical assays, or matrix-assisted laser desorption-based approaches for rapid identification. Thus, developing MGB-specific detection technologies is crucial.

## GENOME UPDATE

PacBio single-molecule real-time (SMRT) sequencing enhances consensus accuracy, particularly in repetitive regions. Consequently, long-read technologies, such as PacBio SMRT and Oxford Nanopore Technology (ONT),produce high-quality bacterial genome assemblies with fewer contigs compared to the conventional method. However, a notable concern with long-read amplicon sequencing is its higher error rates and potential data reliability issues. These challenges, though significant, can often be mitigated by employing specialized assembly techniques (68) or double confirmed using cost-effective Illumina sequencing reads.

The PacBio HiFi sequencing protocol stands out by generating highly accurate long-read data sets, with average read lengths of 10–25 kb and accuracy exceeding 99.5% (69). This approach results in assemblies with significantly fewer errors, both at the single nucleotide level and in small insertions or deletions compared to assemblies produced by ONT (70).

The genome of MPH ASIOC01, sequenced with PacBio HiFi, is currently the only MGB genome with a complete assembly in a single contig. Since 2014, seven MGB genomes have been made publicly available (Table 2). Notably, three of these genomes, MPH ASIOC01 (6), MGB sp. SLW1 (4), and MPH strain PSU-3885-11 (57), were newly deposited in 2024. Among these, MPH ASIOC01 is designated as the reference genome for MPH and holds the largest genome size among MGB species, with a length of 5,765,145 bp (Table 2).

Despite these advances, the limited number of MGB genomes from diverse sources constrains our ability to verify phenotypic variations and to thoroughly study their phylogenetic evolution, which complicates feature comparison. To address this limitation, metagenome-assembled genome (MAG) approaches, which reconstruct genomes from metagenomic data, may provide a promising solution.

## PHENOTYPE AND GENOTYPE OF MGBs

### Glycerol degradation

The biodiesel industry has expanded considerably due to efforts aimed at achieving carbon-neutral mobility in the transportation sector. However, this growth has resulted in a surplus of glycerol as a byproduct, leading to a significant decline in its market value (71). Glycerol-utilizing microbes offer a sustainable route for converting excess glycerol into valuable chemical intermediates and products. MPH ASIOC01 is capable of thriving in epichlorohydrin wastewater, which contains glycerol, 1,3-DCP, and 3-MCPD. This adaptability is likely due to its efficient glycerol degradation system (6).

**Fig 2:**
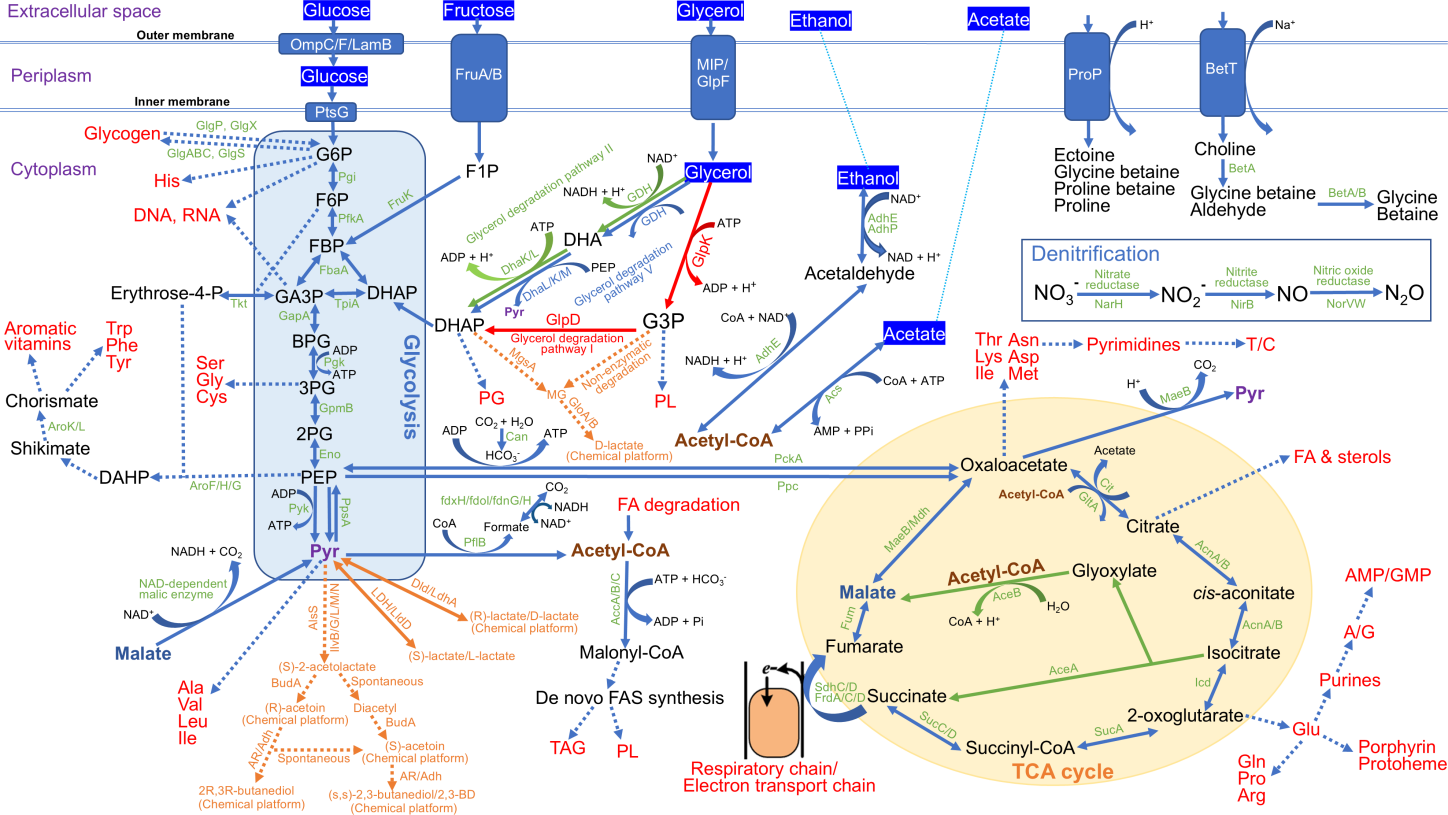
Common biochemical pathways observed in MGBs. This figure was modified from Chin et al. (6).

The system involves key metabolic pathways, including the citric acid cycle, glycerol degradation pathways I, II, and IV, gluconeogenesis I, and glycolysis III (Fig. 2). Our studies have confirmed that MPH ASIOC01 can utilize glycerol as its carbon source. Interestingly, all available MGB genomes display similar glycerol degradation pathways (Table 3), indicating that glycerol utilization is a conserved trait among MGBs. Therefore, it may be possible to promote the growth of other MGB strains using glycerol or its derivatives as their carbon sources.

**TABLE 3:** Phenotype and genotype of MGBs

Phenotypes	Features	Proteins or enzymes (from genes^*a*^)
Carbon utilization	Glycerol	GlpF, GlpD, GlpK
Carbon utilization	Glucose	OmpC, LamB (maltoporin), PtsG, MalE, MalF, MalG, MalK, MglA, MglB, MglC
Carbon utilization	Fructose	FruA, FruB, FruK
Carbon utilization	D-xylose	XylA, XylB, XylF
Carbon utilization	Cellobiose	CelB, PTS lactose/cellobiose transporter subunit IIA
Carbon utilization	D-mannitol	MtlD, MtlR
Carbon utilization	Trehalose	TreA, TreB, TreC, TreR, OstA (LptD), trehalose-6-phosphate phosphatase (TPP)
Carbon utilization	Maltose	MalE, MalF, MalG, MalL, MalK, MalM, MalP, MalQ, MalT, MalX, MalZ, LamB, TreB
Carbon utilization	Melibiose	MelB, MelR
Carbon utilization	D-mannose	ManA, ManB (CpsG), ManX, PTS mannose/fructose/sorbose transporter
Carbon utilization	Arabinose	AraA, AraB, AraC, AraD, AraG, AraH
Carbon utilization	Lactose	PTS lactose/cellobiose transporter subunit IIA, beta-galactosidase
Carbon utilization	Rhamnose	RhaA, RhaB, RhaD, RhaM, RhaR, RhaS, RhaT
Carbon utilization	Sucrose	Sucrose-specific PTS transporter subunit IIBC, sucrose-6-phosphate hydrolase, TreB
Carbon utilization	Ribose	RbsA, RbsB, RbsC, RbsD, RbsK, RbsR
Carbon utilization	Galactose	GalE, GalF, GalK, GalM, GalR, GalS, GalT, GalU, MglA, MglB, MglC
Carbon utilization	Sorbitol/glucitol	GutB (SrlB), GutM, GutQ, SrlD
Carbon utilization	Raffinose	Produce acid using raffinose, but no related genes were found in the genomes (1–3)
Carbon utilization	Salicin	Produce acid using salicin, but no related genes were found in the genomes (1–3)
Carbon utilization	Ascorbic acid	UlaD, YiaK, PTS ascorbate transporter subunit IIC
Carbon utilization	Acetate	Acs
Carbon utilization	Ethanol	AdhE, AdhP
Carbon utilization	Inositol (only in MGB sp.) MFB070)	IolB, IolC, IolD, IolE, IolG, IolH, IolR, IolT (71) MPH MP23^T^ can produce acid with inositol (1)
Carbon utilization	Citrate	CitC, CitD, CitE, CitF (CitC, CitD, CitE, CitF not found in MYI)
Endophytic feature	Cellulose degradation	BcsZ, BglX, BcsZ (BcsZ was not found in MPH PSU-3885-11)
Endophytic feature	Nitrogen fixation (NF)	NifA, NifB, NifD, NifE, NifH, NifJ, NifK, NifL, NifL, NifM, NifN, NifS, NifU, NifV
Endophytic feature	Inorganic phosphate solubilizing (iPS)	Ppx, Ppa, Gcd
Endophytic feature	Organic phosphorus (P_o_) mineralizing	PhoA
Endophytic feature	Phosphate transporter	PitA, PstA, PstB, UgpQ
Endophytic feature	Phosphate intake regulatory	PhoB, PhoR
Endophytic feature	Potassium solubilization (KB)	AckA, Mdh, Ppc, DcuA, DcuC, CitT, DctP
Endophytic feature	Iron sequestration	Enterobactin (tris-catecholate siderophore)
Endophytic feature	Type VI secretion system (T6SS)	VgrG, Hcp, ClpV (TssH), RbsB
Metal homeostasis	Iron	FeoA, FeoB, FeoC, FepB, FetA, FetB, non-heme–ferritin-like protein, bacterioferritin
Metal homeostasis	Copper	CutC, CutF (NlpE), CopA
Metal homeostasis	Copper (only in MYI and MGB. sp. SLW1)	Copper resistance protein B, CopC, CopD
Metal homeostasis	Zinc	ZnuA, ZnuB, ZnuC, Zinc/cadmium/mercury/lead-transporting ATPase
Metal homeostasis	Mercury (only in MPH ASIOC01)	MerA, MerC, MerP, MerR
Metal homeostasis	Mercury	Zinc/cadmium/mercury/lead-transporting ATPase
Metal homeostasis	Nickel and cobalt	Rcan (nickel/cobalt efflux protein), RcnB (nickel/cobalt homeostasis protein), nickel/cobalt transporter, HoxN, HupN, NixA family nickel/cobalt transporter
Metal homeostasis	Magnesium and cobalt	MgtA, MgtE, CorA, CorC, MntH, MntP, MntR, MntS
Metal homeostasis	Cadmium	MntH
Metal homeostasis	Divalent metal cation	MntH, zinc/cadmium/mercury/lead-transporting ATPase, CutA
Arsenate reduction	Arsenic	ArsC
Nitrogen metabolism	Denitrification	NirB, NarH, NorV, NorW (NarH, NorV, and NorW not found in MYIs and MGB sp. MFB070)
Bioremediation	Cr reduction	NfsA, NfsB, NemA (N-ethylmaleimide reductase)
Bioremediation	Dye degradation	AzrG (FMN-dependent NADH azoreductase)
Bioremediation	Breakdown of benzoate compound (possible)	Nitrilase, 4-carboxymuconolactone decarboxylase, acetyl CoA acyltransferase, acetyl dehydrogenase, 4-oxalocrotonate tautomerase, and 4-oxalmesaconate hydratase (4)
Bioremediation	Aminobenzoate metabolism	(S)-mandelate dehydrogenase, 4-hydroxybenzoate decarboxylase subunit C, gallate dioxygenase, and gallate decarboxylase subunit C (4)
Biofilm formation	Colanic acid (CA)-based biofilm	CA synthesis gene cluster, PmrA, PmrB, RcsA, RcsB, RcsC, RcsD, RcsF, ArnT (PmrK), PagP
Motility	Flagellar biosynthesis proteins	FlhA, FlhB, FliR
Motility	Basal body rod proteins	FlgB, FlgC
Motility	Assembly and motor switch protein	FliH, FliG, FliM, FliN
Motility	Stator	MotA, MotB
Motility	L, MS and P ring protein	FlgH, FliF, FlgI
Salt tolerance	Solute production/transportation	ProA, ProB, ProP, ProV, ProW, BetA, BetB, BetT, UgpA, UpgB, UgpC, UgpE, MalK
Acid resistance	Survival in wide pH range	ArgR, AspA, IlvE, Cfa, DnaK
Cellulose biosynthesis	Bacterial cellulose	BcsA, BcsB, BcsC, BscD, BcsE, BcsF, BcsG, BcsO, BcsQ, DgcQ (BscC, BcsE, BcsF and BcsG were not found in MPH PSU-3885-11)
Chemical platform	D-lactate	GloA, GloB, Dld (Dld not found in MGB sp. SLW1)
Chemical platform	Acetoin and 2,3-butanediol	Acetoin reductase (AR), Ilv genes, (AlsS and BudA only found in MPHs)
Drug resistant	Multidrug-resistant cluster	MdtABC-TolC, MdtD, MdtH, MdtK (novobiocin and deoxycholate resistance)

^*a*^ Genes detected in the genome of MPH ASIOC01, MPH PSU-3885-11, MPH MP23^T^, MPL MSSRF40^T^, MYI SaN21-3, MGB sp. MFB070, and MGB sp. SLW1.

### Carbon source utilization

MGB strains exhibit versatile metabolic capabilities, enabling them to utilize a wide range of carbohydrates. The enzymes responsible for incorporating carbon sources, such as glycerol, glucose, fructose, acetate, and ethanol, into the primary metabolic pathways of MGBs are illustrated in Fig. 2. Our prior research has demonstrated that MPH ASIOC01 can utilize these carbon sources (6). Genome analyses suggest that other MGB strains may possess similar metabolic flexibility, although further experimental validation is required.

Genes involved in the metabolism of carbon sources, such as glycerol, glucose, fructose, D-xylose, cellobiose, D-mannitol, arabinose, lactose, rhamnose, sucrose, ribose, galactose, sorbitol/glucitol, ascorbic acid, acetate, ethanol, and citrate, are commonly found across most MGB genomes (Table 3). Notably, MGB sp. MFB070 has acquired a unique cluster of inositol-degrading genes, which are absent in other MGB strains (72). Additionally, the phypat + PGL predictor indicated that MGB sp. SLW1 can utilize acetate (4), aligning with an earlier report showing that MPH ASIOC01 can use acetate as a sole carbon source (6).

In mangrove ecosystems, complex carbon sources primarily originate from the high carbohydrate content found in leaves and roots. These carbon-rich environments support thriving populations of MBG strains. The flexible carbon metabolism of MGBs also allows them to adapt to a variety of other environments. Furthermore, symbiotic bacteria play essential roles in the carbon mineralization process within mangrove ecosystems, though many of these symbionts and their contributions have yet to be fully characterized.

### Metal and arsenic hemostasis

The bacterial genome serves as a reservoir for various heavy metal and metalloid resistance gene clusters. MGB genomes contain genes involved in the homeostasis of iron, copper, zinc, mercury, nickel, cobalt, magnesium, cadmium, and divalent metal cations (Table 3). Notably, MPH ASIOC01 has acquired the *merA*, *merC*, *merP*, and *merR* genes for mercury homeostasis, which are absent in other MGB genomes.

MGB genomes exhibit broad-spectrum resistance to other metals. MGBs encode the *mdtABCDHK* multidrug-resistant cluster, which enhances resistance to novobiocin and deoxycholate, as well as to heavy metals such as cobalt, cadmium, zinc, arsenic, and copper. Genes involved in iron acquisition, specifically tri-catecholate siderophores such as enterobactin, were identified in MGB genomes. The presence of the enterobactin ABC transporter system likely plays a role in iron (FepB) uptake and assimilation, influencing plant growth in Sundarbans mangrove vegetation (4). The presence of corC, which encodes a magnesium and cobalt efflux protein, in all MGB genomes suggests a role in Co²^+^ resistance (Table 3). MGB genomes also contain the gene for arsenate reductase (ArsC), responsible for converting As⁵^+^ to the more toxic As³^+^ (Table 3).

The broad range of metal resistance genes in MGB genomes suggests that the collection site may act as a sink for heavy metals, likely influenced by anthropogenic activities. The study of MGB sp. SLW1 and other MGB strains opens the door to exploring their potential as biomonitoring tools for long-term tracking of ecological health in coastal ecosystems (4) or bioremediations of specific environmental wastes.

### Cellulose metabolism

The genome annotation has revealed that all MGBs possess genes encoding cellulase and enzymes involved in cellulose biosynthesis, suggesting that MGBs could function as cellulotrophs (Table 3). Recently, two novel MGB isolates, strains Eve2-HG and Nin4-HG, have demonstrated cellulose digestion capability (55). For these novel MGB isolates (Eve2-HG and Nin4-HG), it is unclear if their cellulose digestion capability was determined via *in vitro* assays, genome analysis, or some other method. Specifying this would improve scientific rigor. The genomes of MGBs encode four enzymes: α-glucosidase, glycosyl hydrolase family 8 (endoglucanase, cellulase family D), cellulose synthase complex periplasmic endoglucanase, and glycosyl hydrolase family A (cellulase family A), which are involved in cellulose degradation. These enzymes play essential roles in breaking down cellulose, particularly cellulases and endoglucanases, which are crucial for enabling endophytes to penetrate plant roots (73). The ability of MGBs to degrade cellulose likely explains their prevalence in high-fiber fermented foods like kimchi (40) and mandai (41). Substrate annotation via dbCAN3 provides further insights into the utilization of substrates, such as pectin, starch, cellulose, galactan, and alginate, by MGB sp. SLW1 (4).

MGBs contain a complete *bcs* gene cluster for cellulose biosynthesis, as shown in Table 3. This comprehensive array of cellulose biosynthesis proteins likely facilitates the synthesis of bacterial cellulose (BC), which possesses notable physicochemical properties, including porosity, mechanical strength, elasticity, transparency, high polymerization, nanostructure, purity, water retention capacity, biodegradability, and biocompatibility. Moreover, BC is non-cytotoxic and non-genotoxic, making it valuable across various industries (74). The production of BC by MGBs using glycerol is economically feasible. Interestingly, some genes involved in cellulose metabolism were absent in the genome of the MPH strain PSU-3885-11, isolated from a human sputum. This suggests that cellulose metabolism-related genes may have been lost or altered during the transmission of MGBs from environmental or food sources to human hosts, highlighting possible niche-specific genomic adaptations.

### Endophytic features

MGBs are considered as endophytes because of their ability to uptake nutrients, NF, and response to oxidative stress (75). Endophytes can live within or on the surface of plant tissues and often provide benefits through complex mechanisms, including the solubilization of nutrients in the rhizosphere. Key endophytic features of MGB include the presence of cellulose breakdown enzymes and the type VI secretion system (T6SS), along with their abilities in NF, inorganic phosphate solubilization (iPS), organic phosphate (P_o_) mineralization, metal solubilization, iron sequestration, and potassium solubilization (KS) (see Table 3).

MGBs are NF bacteria that thrive in a nitrogen-free environment (3, 29). MYI TULL-A^T^ (2), MPH MP23^T^ (1), MPL MSSRF40^T^ (3), and MGB sp. MFB070 (20) are known as NF bacteria. MGB genomes harbor a complete set of *nif* genes responsible for NF, making it a conserved trait among all MGB strains.

All MGBs possess the T6SS, a protein secretion mechanism that is common in endophytes and plays key roles in facilitating plant–microbe interactions (76). The T6SS is crucial for the survival and fitness of bacteria (77).

The phosphorus cycle in MGBs is facilitated by several key genes. These include those involved in iPS such as *gcd* (gluconate dehydrogenase), *ppa*, and *ppx*. Additionally, genes for P_o_ mineralization (e.g., *phoA* and *phoD*) and phosphate transport (e.g., *pit*, *pstA*, *pstB*) were also identified. These observations confirm that MGBs can solubilize, import, and metabolize phosphate from their environment. PSB has garnered significant attention recently for their potential to enhance plant growth and development. They influence phosphorus availability through microbial decomposition of organic matter, releasing phosphorus from organic sources for plant uptake, or by producing enzymes that enhance phosphorus absorption in plants (78).

The identification of polyphosphate (polyP) kinase 1 in MGB genomes (Table 3), which is responsible for polyP accumulation, underscores its critical role in phosphate storage. Notably, polyP accumulated when MPH ASIOC01 was propagated in high-salinity growth media containing 5% NaCl (65). PolyP is linked to microbes' ability to withstand physical and chemical stresses, playing a crucial role in their adaptation to extreme environments (79). This ability to accumulate polyP may have important applications in developing stress-resistant bacterial strains for use in agricultural biotechnology, particularly in enhancing plant resilience in high-salinity soils.

KS endophytic bacteria function by synthesizing and releasing organic acids. These acids dissolve insoluble minerals, facilitating the release of soluble potassium from the previously mentioned sources (80). Malic acid, citric acid, fumaric acid, gluconic acid, lactic acid, succinic acid, and formic acid were identified in MPH ASIOC01, along with enzymes involved in their synthesis and transport, such as AckA, Mdh, Ppc, DcuA, DcuC, CitT, and DctP, found in the genomes of all MGBs. This indicates that MGBs are KS bacteria.

To date, only MPH has been officially documented as an endophyte (1). Interestingly, the endophytic genes were found in all MGB genomes (Table 3), including the zoonotic MPH strain PSU-3885-11. However, the role of these genes in zoonotic MGBs remains unclear.

### Bioremediation features

MPH ASIOIC01 (6) and the MYI strain T1 (China patent CN113913329B) have both demonstrated the ability to reduce COD, xenobiotics, and organic pollutants (OPs). MGBs were notably detected in numerous wastewater treatment plants (WWTPs) and industrial effluents (Table 1). MGBs were also found in environments contaminated with hydrocarbons (HC), such as biodiesel tanks, water from natural oil-emitting lakes, and HC-extraction systems (Table 1). These observations suggest its ability to bioremediate xenobiotics, especially OPs and HCs. The genome of MGB sp. SLW1 encodes several enzymes, such as nitrilase, 4-carboxymuconolactone decarboxylase, acetyl CoA acyltransferase, acetyl dehydrogenase, 4-oxalocrotonate tautomerase, and 4-oxalmesaconate hydratase genes, putatively indicating the ability to break down benzoate compounds (4). MGB sp. SLW1 and MPH ASIOC01 both possess partial genetic information for degrading naphthalene, styrene, toluene, xylene, and atrazine. This broad range of xenobiotic breakdowns suggests that the Sundarbans mangrove and activated sludge (AS) systems are at risk from various pollutants. This is an insightful point that adds depth and invites future research but could be expanded with specific suggestions for areas needing more study. The genomic analysis of MGB sp. SLW1 and MPH ASIOC01 revealed a wealth of metal and metalloid resistance genes, xenobiotic degradation pathways, and biosynthetic gene clusters, highlighting their ability to survive in harsh environments and their metabolic capabilities to degrade aromatic compounds in WWTPs.

Nasrin et al. (17) proposed that the MYI strain AKS2 can break down Basic Red-18 dye (17). This cationic azo dye is commonly used for textile coloring but is associated with toxicological properties, including carcinogenic and mutagenic effects. Despite this, the use of acid dyes containing azo groups remains widespread in the leather and tannery sectors (81). Azoreductases are enzymes capable of facilitating the degradation of azo dyes. These enzymes are widely used in the pharmaceutical, food, cosmetic, and textile industries. They catalyze the reductive cleavage of azo bonds (–*N* = N–), resulting in the formation of colorless aromatic amines (82). FMN-dependent NADH azoreductase (AzrG) is present in the genomes of all MGBs (Table 3), indicating their potential role in the bioremediation of azo dyes in textile and tannery effluents (15, 16).

Cr(VI) is recognized as hazardous waste and requires proper treatment before disposal. Recent studies have shown that MYI and MPL can effectively carry out the bio-reduction of Cr(VI) (15, 16). Previous studies have indicated that nitroreductases, specifically NfsA and NfsB, derived from *Vibrio harveyi* and *Escherichia coli* (83), along with N-ethylmaleimide reductase (NemA) from *E. coli*, exhibit significant efficacy as chromate reductases (84). The presence of the *nfsA*, *nfsB*, and *nemA* genes in the genomes of MGBs (Table 3) suggests that all MGBs possess the ability to reduce Cr(VI).

### Other specific features of MGBs

Key enzymes for denitrification, including nitrate reductase (NarH), nitrite reductase (NirB), and nitric oxide reductase (NorV/W), were annotated in all MGB genomes (Table 3; Fig. 2). Therefore, MGBs could function as denitrifying polyP-accumulating organisms if introduced into AS systems. All MGBs possess a conserved colonic acid synthesis gene cluster and an assembly system for biofilm formation (Table 3). Biofilm production is another recognized strategy for microbial survival under stress conditions (85). MGBs appear to utilize osmosensing transporters like ProP to detect and respond to varying osmotic pressures. They possess conserved proteins such as ProB, ProV, BetA, and BetB, among others. These proteins may help in the import and synthesis of osmoprotectants such as proline, glycine, and betaine. By mediating osmoregulatory responses, osmosensors aid MGBs to survive in fluctuating osmotic conditions, allowing them to thrive in environments with salinities of up to 20% (86).

Most MGBs possess enzymes for synthesizing chemical platforms, including D-lactate (via GloA, GloB, and Dld) and acetoin and 2,3-butanediol (via acetoin reductase, Ilv proteins, AlsS, and BudA) (Fig. 2; Table 3). Lactic acid is an important platform chemical used to produce various compounds including polylactic acid (87). Acetoin (3-hydroxy-2-butanone) is widely used in food and cosmetics industries as a taste and fragrance enhancer. It can also be utilized as a building block for the synthesis of valuable chemicals such as acetylbutanediol and alkyl pyrazines (88). 2,3-Butanediol has significant potential in industries such as chemicals, cosmetics, agriculture, and pharmaceuticals. However, its industrial production is constrained by the high costs of petro-based methods (89). By utilizing inexpensive carbon sources, like glycerol, and exhibiting traits like rapid growth under high salt stress, a flexible pH range, and resistance to various metal toxicities, MGBs offer promising prospects for large-scale chemical platform production.

## FREE-LIVING MGBs

Free-living MGBs are present in diverse environments, including AS from WWTPs, tannery effluents, and mangrove litterfall, among others (Table 1). Key features that allow MGBs to survive include their diverse ability to utilize various carbon sources and their capacity for metal homeostasis, which helps them resist metal toxicity in different habitats. Their biodegradation capabilities make them excellent candidates for inclusion in bacterial consortia for effective bioremediation. WWTPs located near the coast and at sea level often face challenges related to seawater intrusion (90). Seawater intrusion, along with its deliberate use in chemical production, may facilitate the spread of MGBs from coastal waters into WWTPs.

MGBs were enriched in specific habitats; for instance, a significant prevalence was observed in high-salt sludge (11), aerobic granular sludge (AGS) with acyl-homoserine lactone introduction (8), and during the early phase of halotolerant aerobic granulation for AGS formation (9). In these sludges, MGBs represented 45.7%, 35.9%, and 74.9% of the total microbiome abundance, respectively (Table 1).

The observed high prevalence of MGBs, particularly during the initial phases of AGS development, suggests that they may serve as primary colonizers (91). MGBs may play a crucial role in supporting biofilm formation and modification, as well as in modulating surface characteristics, thereby facilitating the colonization of diverse species in AS systems. Monitoring the population dynamics of MGBs periodically is essential for understanding the factors behind changes in abundance; thus, MGBs may serve as environmental sensors.

According to Li et al. (9), during Phase I of salt-tolerant AGS formation, MGBs, known for their ability to secrete extracellular polymeric substances (EPS), were predominant in forming initial aggregates, accounting for 74.9% of total abundance. However, by the end of Phase III, their abundance sharply declined to 4.2% (9). This suggests that MGB populations will not remain consistently low; they can periodically resurge upon re-entering the dominance stage.

Building upon this understanding, Fujita et al. (22) further highlighted dynamic changes in microbial community structure during incubation. The community eventually reached a quasi-stable state, dominated by several genera, including MGBs. In this simplest network structure, MGBs provide arginine and ethanol to other bacteria, such as *Hydrotalea*, *Terracidiphilus*, and *Rhizomicrobium*. Notably, despite lower abundance in the community, MGBs can exert a disproportionately large impact on the dynamics of the entire microbiome (22).

Li et al. (8) illuminated methods for enriching MGBs within mixed populations. They found that increasing salinity enhances MGB populations in AGS. At 4% salinity, the microbial community was predominantly composed of MGBs, *Propionibacterium*, and *Vibrio*, with relative abundances of 26.6%, 24.9%, and 20.4%, respectively. The addition of acyl-homoserine lactone stimulated the secretion of EPS, allowing MGBs to emerge as the dominant genus, with a relative abundance of 35.9% in the salt-tolerant AGS at 4% salinity (8).

MGBs may serve as pioneer colonizers, detoxifying environments to enhance habitability and providing organic carbon and bioavailable nitrogen to subsequent microbial communities (92). However, changes in environmental conditions or the introduction of competing species can lead to a decrease in MGB populations. Monitoring their dynamics through frequent microbiomic analyses could help identify periods of dominance and decline. This variability indicates that MGB population sizes may differ across various sample types, depending on the organic composition of wastewater. Nonetheless, further extensive research is needed to fully elucidate these intriguing observations.

## PLANT-ASSOCIATED MGBs

### MGBs as endophyte

MGBs exhibit endophytic characteristics, forming symbiotic relationships with plants. MGBs have been documented to form associations with various plant species, including *Phragmites karka*, *Spartina alterniflora,* and *Avicennia marina*, among others (Table 1). MPH strain MP23^T^ has been identified as an NF strain, which grows on nitrogen-free agar and the production of 0.294 mg of total nitrogen per mL of broth after 5 days of incubation at 30°C (1). Although MGBs have been identified as endophytic bacteria based on their isolation sources (Table 1) and genetic evidence (Table 3), there is a lack of studies investigating these plant–microbe interactions. Currently, insufficient experimental data exist to determine whether MGBs are exclusively endophytic or pathogenic. The potential benefits of MGBs for plant hosts, such as growth enhancement through pathogen inhibition, increased auxin production, improved nutrient absorption, greater stress tolerance, and enhanced disease resistance, remain largely unexplored, warranting further investigation into their roles in plant health and ecosystem dynamics.

### MGBs play a role in agriculture and food products

Increasing experimental evidence highlights the association of MGBs with agricultural products, which ultimately find their way into our daily food chain. For instance, MGBs have been detected in the pericarps of mangosteen (*Garcinia mangostana*) and pigeon pea (*Cajanus cajan*) (Table 1). They are also present in various fermented foods and spoiled products, including coffee beans, soy sauce mash, brined olives, pre-fermented coconut water, kimchi, and mandai, a fermented food made from the inner skin of jackfruit. MGBs have been found in spoiled green manzanilla Spanish-style table olive fermentations and during the spontaneous decay of açaí palm (*Euterpe oleracea*) fruits (see Table 1). The benefits or impacts MGB may have on food quality, such as aroma, flavor, or food safety, remain unknown.

MGBs are closely associated with food and fermented food products that possess high fiber content, often involving brining or fermentation processes that utilize significant salt concentrations. They contain enzymes, such as cellulases and endoglucanases, that enable cellulose degradation (Table 3), potentially allowing them to utilize the fiber in these products as a carbon source. It is understood that brined olives contain 8%–14% salt, kimchi contains 2%–3% salt, and mandai contains 5%–10% salt. The halotolerant properties of MGBs enable their survival in these high osmotic conditions. Also, fermented products typically harbor large populations of *Lactobacillus* (41, 93). Notably, MGBs can coexist with *Lactobacillus* at low pH levels without inhibition. These microbial interactions could be beneficial, and they would provide insight into the ecological dynamics within the fermented foods.

The ability of MGBs to thrive in processed foods may be attributed to their flexible utilization of carbon sources, broad tolerance to osmotic pressure and pH levels, and capacity for lactic acid metabolism. To enhance the detection of MGBs in fermented foods, incorporating an enrichment step using antibiotics, such as novobiocin, is recommended (40). It would also be intriguing to explore the presence of MGBs in other high-fiber vegetables, fruits, and fermented foods, such as sauerkraut, suan cai (Chinese sauerkraut), and jangaji (pickled vegetables), produced under elevated salinity or low pH conditions.

## ZOONOTIC-BASED MGBs

### MGBs in animals and invertebrates

Many MGBs have been identified as symbiotic bacteria across various organisms in the animal kingdom. These include brachycephalic dog breeds, great tits (*Parus major*), broilers, domestic chickens (*Gallus gallus domesticus*), calves, buffalo, juvenile largemouth bass (*Micropterus salmoides*), and brown-marbled groupers (*Epinephelus fuscoguttatus*), as well as in humans (see Table 1). MGBs have also been detected in a wide range of invertebrates, including the giant mealworm beetle (*Zophobas atratus*), Comal Springs riffle beetle (*Heterelmis comalensis*), African palm weevil (*Rhynchophorus phoenicis*), black soldier fly (*Hermetia illucens*), yellow mealworm (*Tenebrio molitor*), Jamaican field cricket (*Gryllus assimilis*), shrimp, pine wood nematode (*Bursaphelenchus xylophilus*), *Euryphorus nordmannii*, Tioman crab (*Neosarmatium indicum*), and violet vinegar crab (*Episesarma versicolor*) (see Table 1).

Metagenomic analyses have revealed that MGBs can dominate the gut microbiome. The gastrointestinal tract of superworms fed with polyurethane (PU) showed a significant predominance of MGBs, which comprised an impressive 21% of the overall gut microbiota (42, 94). MGBs in the superworms’ guts are also recognized for their association with the degradation of polyvinyl chloride (PVC) (43). The genes responsible for metabolizing these hydrocarbon polymers remain largely uncharacterized, representing a critical gap in current research. In contrast, MGBs in largemouth bass fed a methionine-supplemented diet constituted approximately 6% of the gut microbiota (51), while in shrimp, MGBs made up approximately 5% (53). These findings suggest that while MGBs are present across diverse species, their abundance may vary based on dietary or environmental conditions.

### Association of MGBs with humans

Early evidence has suggested the presence of MGBs in humans; however, this association was often overlooked or met with skepticism. For example, MGBs were detected in samples from a urinary tract infection cohort, but these findings were dismissed as plant-associated pathogens and considered false positives (60). With the emergence of new evidence, the correlation between MGBs and humans has become increasingly apparent. MGBs have been detected in human lungs (56), gastric juice (58), feces (59, 62), and tear film (61). Recently, the isolation and whole-genome sequencing (WGS) of the MPH strain PSU-3885-11 from human sputum (Table 2) further substantiate the presence of MGBs in human tissues and organs (57). As of now, no direct association has been established between MGBs and pathogenicity.

Gazi et al. (58) proposed that MGB is one of the gastric bacteria capable of enduring the acidic gastric environment (58). Acid resistance genes present in *Lactobacillus*, including *argR*, *aspA*, *ilvE*, *cfa*, and *DnaK* (95), were also found in all MGBs (Table 3). Since MGBs are commonly found in high-salinity fermented foods and demonstrate resistance to gastric acid, it is likely that they can be transmitted to humans through these food sources. It has been previously reported that brachycephalic dog breeds possess a distinct nasal microbiota that includes MGBs (46). Consequently, the presence of MGBs in the human airway or lung microbiota is not unexpected (56). Despite metagenomic evidence indicating that MGBs can colonize the human gut, lungs, and urinary tract, our understanding of their functional behavior within the human organ microecosystem remains limited.

It is intriguing to explore the potential role of MGBs as a novel form of probiotic carrying biodegradation capabilities in the human gut. Micro(nano)plastics (MNPs) are increasingly prevalent in the food chain, leading to heightened human consumption. Given the evidence of microbial plastic degradation by bacteria and the association of MGBs in the gut microbiota of *Zophobas atratus*, where they contribute to the breakdown of PU and PVC, it raises the question of whether human gut bacteria, particularly MGBs, could participate in the biotransformation of ingested MNPs (42, 43, 94, 96). While no direct evidence currently supports this process in humans, the ability of MGBs to degrade plastics in other organisms suggests this as a fascinating area for further investigation.

Finally, the aryl polyene (APE) biosynthetic gene pathway (Region 3) was present in the genome of MGB (6). Host-associated bacteria, encompassing both commensals and pathogens impacting humans, animals, invertebrates, and plants, frequently harbor APEs. This discovery bolsters the proposition that MGBs may function as zoonotic or endophytic organisms (Table 1).

## POSSIBLE TRANSMISSION MODE OF MGBs

**Fig 3:**
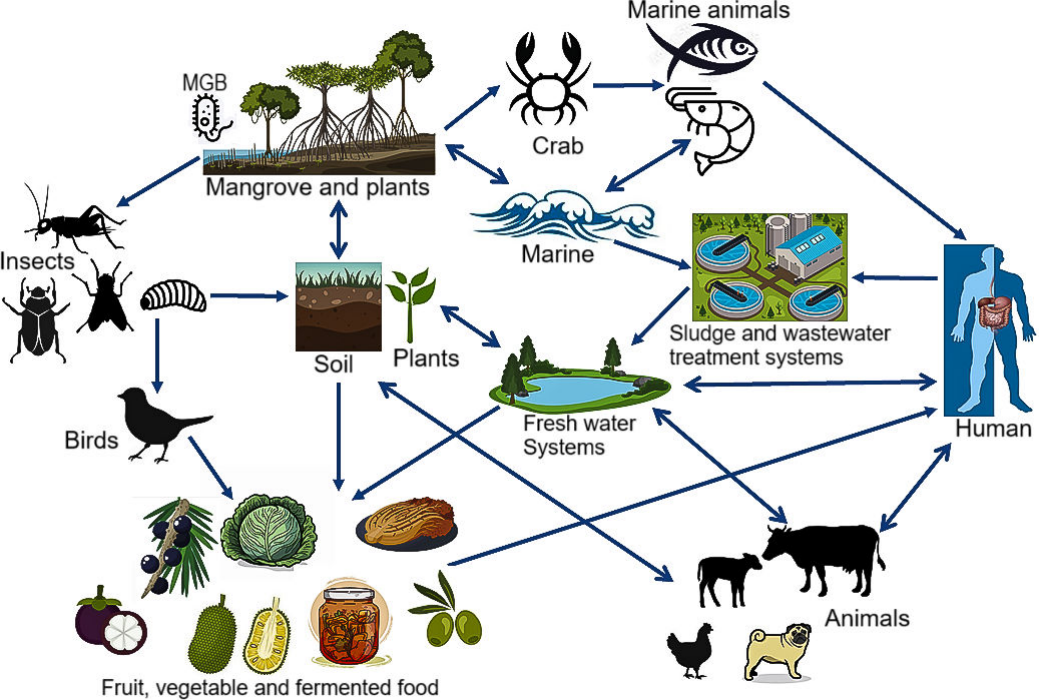
Predicted modes of transmission for MGBs across ecosystems.

A few reports suggest the possible transmission of MGBs between habitats. For instance, numerous cellulolytic bacteria, including MGBs, are commonly found in mangrove sediments and as endophytes in mangrove trees. It has been reported that mangrove sesarmid crabs presumptively acquire horizontally transmitted cellulolytic symbionts to aid in cellulose digestion (55). On the other hand, MGBs have also been detected in the frass of the black soldier fly (*Hermetia illucens*), yellow mealworm (*Tenebrio molitor*), and Jamaican field cricket (*Gryllus assimilis*) (45). Frass, a nitrogen-rich insect excrement used as an organic fertilizer, may serve as a medium for transmitting MGBs from insects to soil and plants. It is noteworthy that the great tit (*Parus major*) exhibited a significant increase in the relative abundance of MGBs when they were fed a mixed diet of mealworms, berries, and seeds. Conversely, switching from this mixed diet to a standard diet of seeds and mealworms resulted in a significant reduction in MGBs in its gut community. This illustrates how dietary fluctuations can lead to changes in bird gut microbiomes (47). Hence, it is reasonable to assume that MGBs can be transmitted to a bird’s gut through the consumption of specific diets. Based on existing evidence, we hypothesize that MGBs may have multiple transmission pathways and circulate across various ecosystems. For instance, MGBs might be transmitted via a marine route from mangroves to mud crabs, then through seawater or marine animals such as fish and shrimp, eventually reaching humans. Another possible route is terrestrial transmission, where MGBs spread from insects or animals to soil or freshwater systems and may eventually reach humans through direct contact with animals or consumption of MGB-containing food. MGBs can be transmitted in both directions: from ecosystems to humans and vice versa. They may also re-enter ecosystems through human activities or WWTP effluent (Fig. 3). However, these proposed transmission modes need to be further validated through more rigorous primary studies including longitudinal, cohort, or case-control studies. It is also worth investigating whether MGBs can undergo vertical transmission, which refers to the transfer of genetic material and microorganisms from parents to their offspring. This type of transmission is common among mammals (97). By analyzing distribution data and the potential transmission pathways of MGBs, hypotheses can be developed to investigate their ecological roles, adaptive strategies, and evolutionary relationships across different habitats. Notably, Chaichana et al. highlight the importance of continuous monitoring of environmental bacteria, such as MGBs, which have the potential to become human pathogens—particularly in immunocompromised individuals (57).

## CONCLUSION AND FUTURE DIRECTION

This minireview offers a valuable roadmap for formulating hypotheses to guide future genetic, biochemical, phylogenetic, and ecological studies of MGBs. Notably, MGBs exhibit resilience to high salinity, extreme pH, and metal toxicity, commonly found in mangroves and coastal areas rich in diverse carbon sources. Future research should focus on elucidating the physiological adaptations triggered by abiotic stresses, including hypoxia, anoxia, and varying carbon source availability. Utilizing omics technologies, such as metabolomics, RNA-seq, and proteomics, presents a promising approach to addressing these questions. A pioneering study by Varghese et al. (98) examined the transcriptomic dynamics and molecular mechanisms of MGB sp. MFB070 in response to hypoxia and anoxia. The study found that the expression of genes encoding aconitase and invertase was upregulated under hypoxic and anoxic conditions, with proline biosynthesis also elevated during anoxia (98). In our earlier report, we employed the Fe-IMAC-LCMSMS approach to investigate the effects of osmotic stress on the phosphoproteome of MPH ASIOC01. We proposed hypotheses regarding the role of polyP as a primordial chaperone that safeguards protein integrity under high-salinity conditions (65). By analyzing changes in metabolites, mRNA, proteins, and their post-translational modifications, we aim to identify both abiotic and biotic factors that contribute to MGBs' ability to thrive under these conditions.

This minireview affirms the association of MGBs with humans. While their functionality in human organs remains unclear, this finding underscores the need for future research into the complex symbiotic or pathogenic interactions between humans and MGBs. Evidence-based research is essential to elucidate plant–microbe interactions, phylogenetic evolution, transmission modes, and the role of MGBs in maintaining microbiome population dynamics across diverse ecosystems. This review emphasizes the potential of MGBs as environmental sensors, bioremediation tools, versatile hosts for chemical platform production, key players in food fermentation, and promising new probiotics for degrading MNPs and other xenobiotics in the human gut.
